# Behaviour of Plate Anchorage in Plate-Reinforced Composite Coupling Beams

**DOI:** 10.1155/2013/190430

**Published:** 2013-10-31

**Authors:** W. Y. Lam, Lingzhi Li, R. K. L. Su, H. J. Pam

**Affiliations:** Department of Civil Engineering, The University of Hong Kong, Pokfulam Road, Hong Kong

## Abstract

As a new alternative design, plate-reinforced composite (PRC) coupling beam achieves enhanced strength and ductility by embedding a vertical steel plate into a conventionally reinforced concrete (RC) coupling beam. Based on a nonlinear finite element model developed in the authors' previous study, a parametric study presented in this paper has been carried out to investigate the influence of several key parameters on the overall performance of PRC coupling beams. The effects of steel plate geometry, span-to-depth ratio of beams, and steel reinforcement ratios at beam spans and in wall regions are quantified. It is found that the anchorage length of the steel plate is primarily controlled by the span-to-depth ratio of the beam. Based on the numerical results, a design curve is proposed for determining the anchorage length of the steel plate. The load-carrying capacity of short PRC coupling beams with high steel ratio is found to be controlled by the steel ratio of wall piers. The maximum shear stress of PRC coupling beams should be limited to 15 MPa.

## 1. Introduction

Plate-reinforced composite (PRC) coupling beam, that is, conventionally reinforced concrete (RC) coupling beam embedded with a vertical steel plate and provided with shear studs for transferring forces between concrete and steel plate, is a practical alternative design to improve the strength, ductility, and energy dissipation ability of conventional RC coupling beams. By adopting this alternative design, the failure mode of coupling beams can be changed from a brittle sliding shear failure at the beam-wall joints to a desirable ductile flexural failure [1]. The experimental results of deep PRC coupling beams subjected to reversed cyclic loading [2] showed that even vertical cracks were formed at the interface between the beam and the adjacent wall piers, with the lateral constraints provided from the surrounding concrete, plate instability was not observed, and plate strengthened beams could still resist very high shear in the postpeak stage.

By considering the transverse and longitudinal slips of the shear studs at the span of the beams, Lam et al. [3] worked out a design formula for determining the number of studs required. By evaluating the bearing stress distribution at the plate anchor, Su et al. [4] developed a design model for the anchor of steel plates in wall piers.

It is well known that laboratory tests are costly and time consuming and, in some cases, can even be impractical due to the limitations of laboratory settings. Recently, Henriques et al. [5] and Ellobody and Young [6] have successfully utilized nonlinear finite element packages to conduct comprehensive investigations on various steel composite structures. Su et al. [7] developed an accurate and efficient nonlinear finite element model to investigate the internal stress and force distributions on the steel plates embedded in PRC coupling beams. In their studies, the finite element models were validated by the well-controlled experimental results before they were used for carrying out the parametric studies. Reliable numerical results, such as full-field internal stress distributions, in far more detail than is possible in laboratory work were obtained. Based on the numerical results, a set of equations for quantifying the shear stud force demands and a series of nondimensional design charts for determining the internal forces of the embedded steel plates were also constructed.

Although extensive effort has been made to determine the arrangement of shear studs and the internal force distribution, the influence of the span-to-depth ratio, the anchorage length, and the steel contents of coupling beams and wall piers on the overall performance of the PRC coupling beams has yet to be studied comprehensively.

Based on the nonlinear finite element model developed in our previous study [7], a parametric study presented in this paper was conducted to investigate the load-carrying capacity of PRC coupling beams and the behaviors of plate anchorage in the wall regions under different combinations of beam geometries, plate geometries, and reinforcement details. The parametric study is proven to supplement the experimental study in investigating beam specimens with strengths (or dimensions) exceeding the capacity (or size) limit of the laboratory settings. Thus a more comprehensive design procedure that takes into account the effects of a wide range of beam geometries and capacities can be obtained.

## 2. Nonlinear Finite Element Modelling

A total of 99 models of prototype PRC coupling beams with different beam geometries were built and analyzed using a nonlinear finite element package ATENA [8]. This paper focuses on a comprehensive investigation on the key parameters which control the overall performance of PRC coupling beams. Thus the choice of member types, the nonlinear finite element modeling, and its verification are just briefly explained. Further details can be found elsewhere [7].

### 2.1. Specimen Details

**Figure 1:**
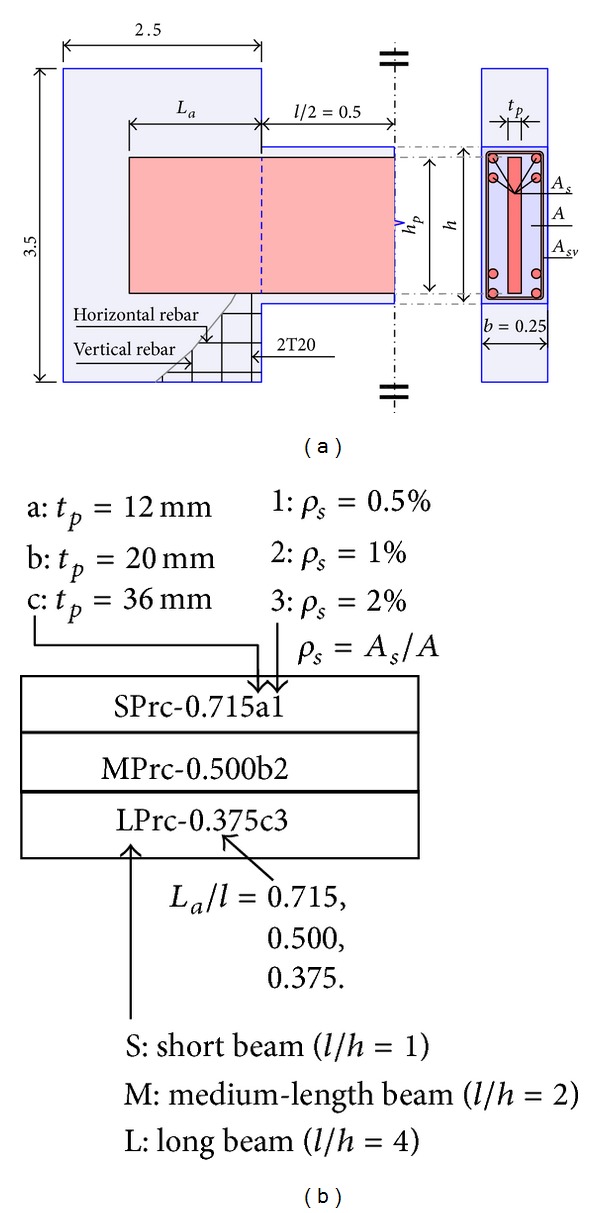
Details of specimens; (a) perspective view and (b) nomenclature (dimensions are in m).

The dimensions of the prototype beams were set within a normal practical range to simulate real coupling beams. To minimize the number of models required, the models were constructed with constant beam lengths (*l* = 1.0 m), wall thicknesses, and beam widths (*b* = 0.25 m), as shown in Figure 1.

**Figure 2:**
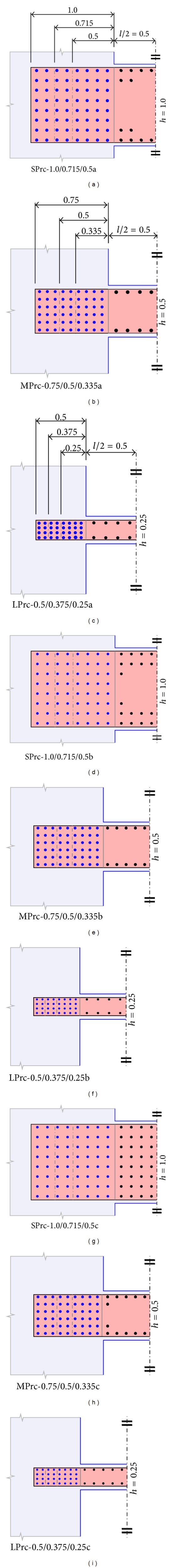
Shear stud arrangements on the steel plates (dimensions are in m).

Paulay [9] and Tassios et al. [10] showed that the failure behaviors of RC coupling beams with different span-to-depth ratios could differ considerably. Therefore, three beam depths (i.e., *h* = 1.0, 0.5, and 0.25 m) were chosen, and the models were divided into three groups, namely, SPrc, MPrc, and LPrc (corresponding to *l*/*h* = 1, 2, and 4, resp., as shown in Figures 1 and 2), so that short (*l*/*h* ≤ 1.5), medium-length (*l*/*h* ≈ 2 to 2.5), and long (*l*/*h* ≥ 4) PRC coupling beams could all be represented and considered in this study.

The required anchorage length (*L*_*a*_) should be determined in conjunction with the shear stud arrangement [3, 4] as

(1) $$\begin{array}{c} L_{a}=2\sqrt{\frac{M_{p}}{w}+\frac{{\left(V_{p}\right)}^{2}}{2w^{2}}}+\frac{V_{p}}{w}, \end{array}$$

where *M*_*p*_ and *V*_*p*_ are the ultimate moment and shear force, respectively, transferred to the steel plate and *w* is the uniformly distributed vertical bearing stress. The value of *L*_*a*_ was varied in each group of models within a practical range that satisfied both of the following criteria concerning the geometry of the plate anchor: 0.25 ≤ *L*_*a*_/*l* ≤ 1 and 0.5 ≤ *L*_*a*_/*h* ≤ 2. Therefore, each group contained three series with different anchorage-to-span ratios as shown in Figures 1 and 2. The values of *L*_*a*_ were varied between 0.5 m and 1.0 m in group SPrc (i.e., *L*_*a*_/*l* = 1.0, 0.715, and 0.5 for SPrc) and between 0.25 m and 0.5 m in group LPrc (i.e., *L*_*a*_/*l* = 0.5, 0.375, and 0.25 for LPrc). The range of *L*_*a*_ was further narrowed down to within 0.335 m and 0.75 m for group MPrc (i.e., *L*_*a*_/*l* = 0.75, 0.5, and 0.335 for MPrc), as the upper and the lower limits of the optimum *L*_*a*_ value for this group were expected to fall between those of groups SPrc and LPrc.

 With the use of concrete with a cube compressive strength *f*_cu_ = 60 MPa, the transverse reinforcement ratio (*ρ*_sv_) was first set to give a theoretical ultimate shear stress in the RC component (*v*_rc_*) of about 6 MPa, which was close to the maximum allowable value of 6.2 MPa given by the new code of practice in Hong Kong [11]. Sufficient longitudinal reinforcement was provided to ensure that the beams could develop their full shear capacities; that is, the beam failures would be governed by shear rather than bending. The plate thickness (*t*_*p*_) in the prototype models was then determined for target theoretical plate shear strength (*V*_p_*) of about 50% of the total theoretical capacity (*V*_u_*). Each basic model was thus provided with three kinds of plate thicknesses (*t*_*p*_ = 12, 20, and 36 mm corresponding to types a, b, and c, resp.) and longitudinal steel ratios (*ρ*_*s*_ = 0.5, 1, and 2% corresponding to types 1, 2, and 3, resp.), as shown in Figure 1 and Table 1. Therefore, the total number of models was 3 groups × 3 series × 3 × 3 types = 81.

The horizontal and vertical wall reinforcement ratios (*ρ*_*wx*_ and *ρ*_*wy*_) were kept constant in each group, with *ρ*_*wx*_ = 1.8% and *ρ*_*wy*_ = 1% in groups SPrc and MPrc and *ρ*_*wx*_ = 1.6% and *ρ*_*wy*_ = 0.9% in group LPrc. Relatively high reinforcement ratios were adopted in the wall piers to ensure that the walls would not fail earlier than the PRC coupling beams in most cases. More horizontal reinforcement was provided because the walls were supported along one of the vertical edges. However, in real engineering practices, as the walls are subjected to very high axial loads, more vertical reinforcement would be provided. As models in group SPrc with 20 mm as well as 36 mm thick steel plates failed prematurely probably due to early failure in the wall regions, *ρ*_*wx*_ and *ρ*_*wy*_ were varied to investigate the effects of wall reinforcement ratio on the development of beam capacities. Thus 18 models for a parametric study on the wall reinforcement ratio (*ρ*_*wx*_ and *ρ*_*wy*_) were added to the 81 models for the parametric studies on the four primary parameters (*l*/*h*, *L*_*a*_/*l*, *t*_*p*_, and *ρ*_*s*_), which made a total of 99 models.

The theoretical ultimate shear stresses (*v*_u_*) were estimated as the lesser of the moment and the shear capacities of the beam, where the moment capacity was calculated from section analysis assuming full plate/RC composite action and the shear capacity was calculated in accordance with the British Standards [12, 13] with the safety factors taken as unity. Details of the method have been presented in [3, 4], and the reinforcement details together with the *v*_u_*s of the prototype PRC coupling beams are listed in Table 1. The shear stud arrangements on the steel plates are depicted in Figure 2. The nominal stud diameter and length were 25 mm and 100 mm, respectively. The characteristic shear strength *Q*_*k*_ of the shear studs, which depends on the concrete strength, was obtained from BS5950 [13]. In order to minimize the required anchorage length, the bearing strengths provided by the shear studs in both vertical and horizontal directions were considered and a maximum number of shear studs was provided in the plate anchors in accordance with the minimum allowable shear stud spacing [13], as shown in Figure 2.

### 2.2. A Brief Introduction to the Finite Element Model

Three- and four-node SBETA elements [8] were used to simulate the concrete in the analysis. The following factors were considered in the nonlinear concrete material model used in the analyses: (1) nonlinear behavior in compression including hardening and softening, (2) fracture of concrete in tension based on nonlinear fracture mechanics, (3) biaxial strength failure criterion, (4) reduction of compression strength after cracking, and (5) reduction of the shear stiffness after cracking (variable shear retention). In order to represent the unique properties of concrete produced in Hong Kong, the initial elastic modulus *E*_0_ and the peak strains *ε*_*c*_ of the local concrete were estimated by the following equations [14], where *f*_cu_ is the cube compressive strength of concrete:

(2) $$\begin{array}{c} E_{0}=6500{\left|f_{\text{cu}}\right|}^{1/3}\left[\frac{\text{MN}}{{\text{m}}^{2}}\right],\\ \varepsilon_{c}=\frac{3.46{\left|f_{\text{cu}}\right|}^{3/4}}{E_{c}}. \end{array}$$

The tensile strength *f*_*t*_′ [15] and fracture energy *G*_*f*_ [16] were defined as

(3) $$\begin{array}{c} f_{t}^{\prime }=0.198f_{\text{cu}}^{2/3}\left[\frac{\text{MN}}{{\text{m}}^{2}}\right],\\ G_{f}=0.000012f_{\text{cu}}^{0.557}\left[\frac{\text{MN}}{\text{m}}\right]. \end{array}$$

Poisson's ratio and compression softening deformation of the concrete were taken as 0.2 and −0.006 m, respectively.

Experimental results obtained by Lam et al. [3] have shown that bond slipping is quite significant for RC coupling beams. The main longitudinal reinforcement of the coupling beams was therefore modeled by the discrete reinforcement model which was able to consider the bond slip effects. The bond-slip relationship of the CEB-FIB model code 90 [17] was used in this analysis.

Each steel plate was modeled using the bilinear steel von Mises model provided in ATENA, where the biaxial failure law was considered in conjunction with the bilinear stress-strain law that took into account both the elastic state and the hardening of steel. A Poisson's ratio of 0.3 was used in considering the biaxial responses of steel plates.

**Figure 3:**
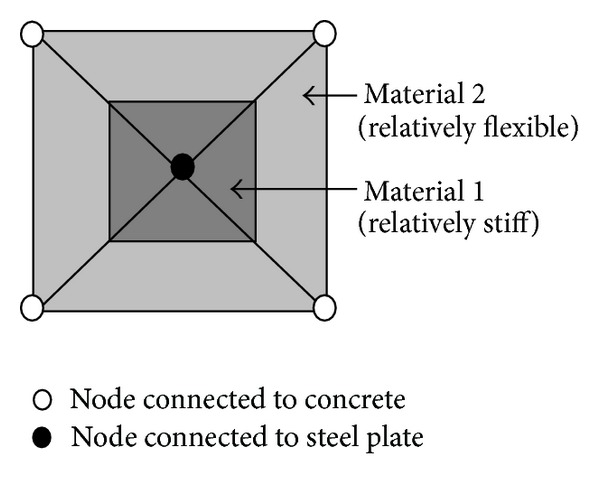
Typical shear stud element adopted in numerical study.

Rectangular shear stud elements with a combination of 4-node quadrilateral and 3-node triangular finite elements (as illustrated in Figure 3) were used to model the shear stud action. The flexible elements with material 2 were introduced as the media for the plate/RC load transfers that allowed for plate/RC interface slips. The elements with material 1 are much stiffer than material 2 and would undergo predominantly rigid body movement only. As a two-dimensional analysis was carried out, where the RC and the steel plate were modeled by two dimensionless layers, only one shear stud element was introduced for transferring loading from the plate to the RC. Thus the element was intended for representing a pair of shear studs, one on each side of the plate, and the bilinear stress-strain relationships of the materials were adjusted to obtain the desired load-slip response for a pair of shear studs.

**Figure 4:**
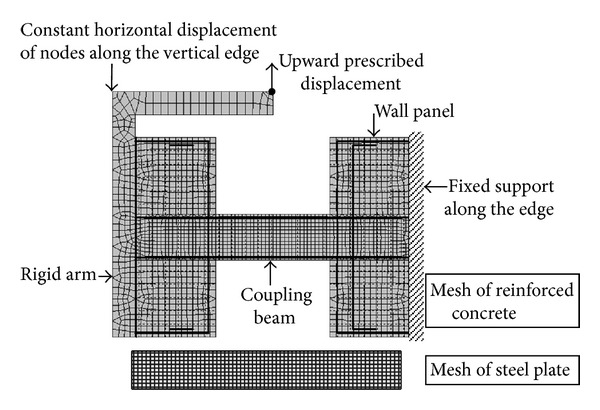
Finite element meshes for modelling PRC coupling beam specimens in NLFEA.

Figure 4 shows the finite element meshes of the nonlinear finite element model of a PRC coupling beam specimen. The concrete was modeled by 4-node isoparametric plane stress finite elements. A fine mesh with element size of about 25 mm was adopted for the steel plate region as it was the main focus in the analysis. The steel plate was modeled by 4-node isoparametric plane stress finite elements of the same size. The locations of the finite element nodes of the steel plate were deliberately set at the centers of the corresponding concrete finite elements to facilitate the introduction of bond and shear stud elements, which would each be connected to a concrete element at its four outer corner nodes and to a plate element node as its center. Smeared reinforcement models were used for the horizontal wall reinforcement, where perfect bond between concrete and steel was assumed in the elements. The beam longitudinal reinforcement as well as the wall vertical reinforcement adjacent to the coupling beam was modeled by 2-node discrete bar elements so as to consider the bond-slip effect as described in the last section. All the nodes along the vertical wall edge on the right were fixed, while the nodes along the vertical wall edge on the left were constrained to undergo equal horizontal displacements. This would maintain parallelism of the two wall panels in the loading process.

**Figure 5:**
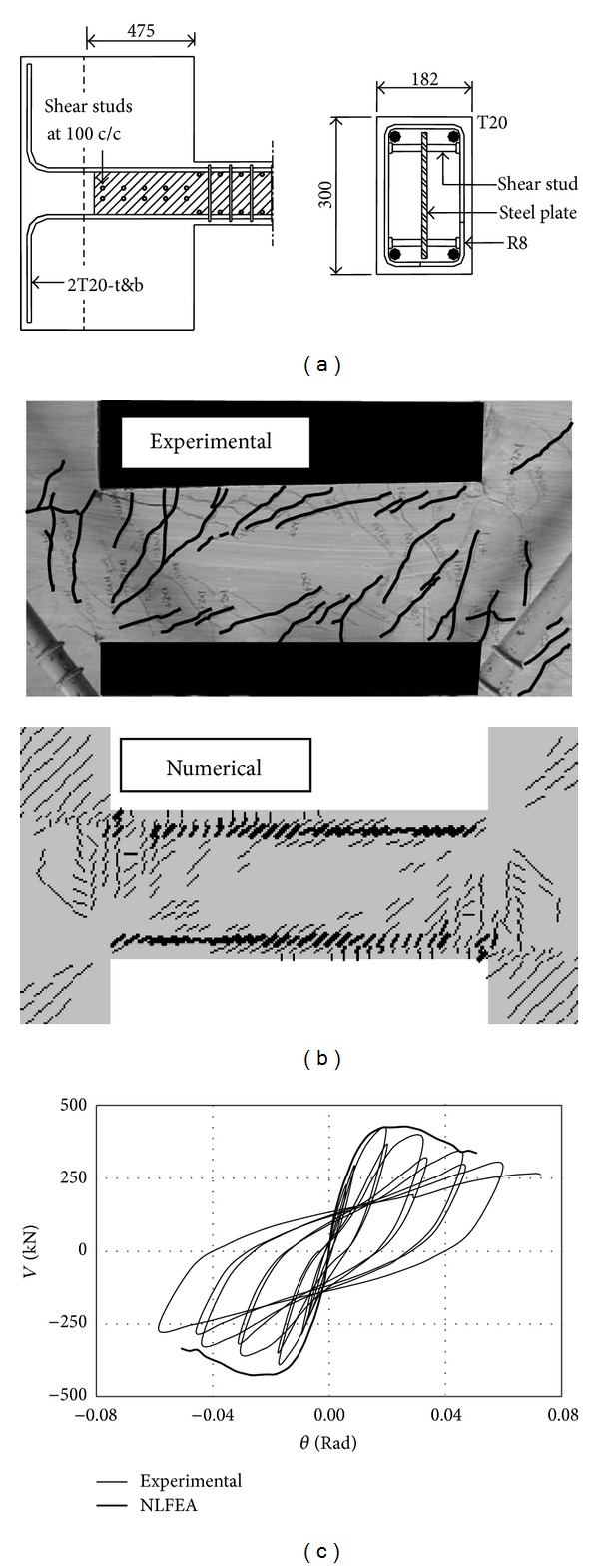
Verification of the numerical model; (a) geometries and reinforcement details of Unit CF, and comparison of numerical and experimental (b) failure patterns and (c) load-drift curves.

Several coupling beams previously tested [3, 4] under reversed cyclic loads with and without embedded steel plate were modeled by the nonlinear finite element model. Only the comparison between the numerical and the experimental results of one of the specimens named “Unit CF” is illustrated in Figure 5, and the further detailed verification can be found in the paper [7]. Figures 5(b) and 5(c) show that the numerical model could accurately predict both the crack pattern and the load-drift response of PRC coupling beams in both elastic and postpeak stages. Thus the nonlinear finite element model could be employed to estimate the strength, stiffness, and ductility of coupling beams.

## 3. Parametric Study

The effects of the variations in (1) span-to-depth ratio (*l*/*h*), (2) plate anchorage length in the wall region (*L*_*a*_), (3) plate thickness (*t*_*p*_), (4) longitudinal reinforcement ratio of beam (*ρ*_*s*_), and (5) wall vertical reinforcement ratio (*ρ*_*w*_) were studied. The material parameters assumed in the study are as follows: *f*_cu_ = 60 MPa (concrete strength), *f*_*y*_ = 460 MPa (deformed bar), *E*_*s*_ = 200 GPa, *f*_*yp*_ = 355 MPa (*t*_*p*_ ≤ 16 mm) or 345 MPa (16 mm <*t*_*p*_ ≤ 40 mm) (grade 50 steel plate), and *E*_*p*_ = 205 GPa.

### 3.1. Effects of Span-to-Depth Ratio

**Figure 6:**
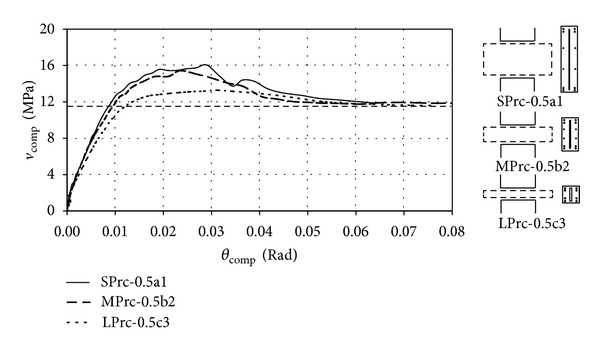
Computed shear stress-drift responses of basic models with *L*_*a*_ = 500 mm.

The effect of span-to-depth ratio on the performances of PRC coupling beams with the same anchorage length of steel plates and roughly the same theoretical ultimate shear is investigated.  Figure 6 shows the computed shear stress-chord rotation (*v*_ comp _–*θ*_ comp _) relationships of three basic models having different span-to-depth ratios. They were all provided with a plate anchorage length of 500 mm (*L*_*a*_/*l* = 0.5) and designed with roughly the same theoretical ultimate shear stress (*v*_u_* ≈ 12 MPa) but different ultimate strengths. All the three models could reach a peak shear stress about 25 to 40% higher than the *v*_u_* value, which was consistent with the experimental results by [3, 4]. Under initial deformations, Units SPrc-0.5a1 and MPrc-0.5b2 could resist similar shear stresses while Unit LPrc-0.5c3 had a weaker response. Unit MPrc-0.5b2 reached a slightly larger maximum shear stress than Unit SPrc-0.5a1, and its strength dropped more steadily than Unit SPrc-0.5a1 after reaching the peak; Unit LPrc-0.5c3 developed the lowest maximum shear stress among the three models but it had the steadiest postpeak strength.

**Figure 7:**
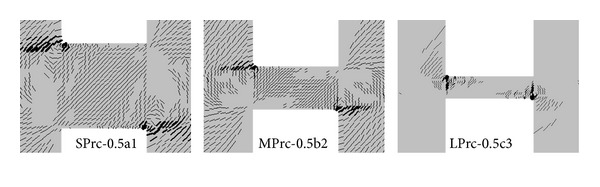
Crack patterns of basic models with *L*_*a*_ = 500 mm at peak loads.

Figure 7 shows the crack patterns of the three models at peak load, where only cracks wider than 0.1 mm are shown. Cracks were only formed in the tension side near each beam end in Unit LPrc-0.5c3 while almost the whole beam was cracked in Unit SPrc-0.5a1. Crack propagations from the beam ends towards the beam center and a transition from the truss action (in Unit LPrc-0.5c3) to the arch action (in SPrc-0.5a1) in the load-resisting mechanism could be observed as the span-to-depth ratio decreased. In Unit LPrc-0.5c3 the failure of the whole beam-wall system was mainly governed by the beam capacity, but the weak component shifted gradually to the walls as the span-to-depth ratio decreased. In Unit SPrc-0.5a1, the walls were cracked almost as seriously as the beam, suggesting that further increasing the beam capacity by increasing the longitudinal steel ratio or the plate thickness could have undesirably resulted in earlier failure in the wall piers than in the beam. In fact, the wall piers in Unit SPrc-0.5a1 were quite heavily reinforced, and it would be rather impractical to further strengthen the wall piers by increasing the wall reinforcement ratio. Thus, there should be an upper limit for the enhanced shear strength of the coupling beam to ensure desirable failure sequence of the structural members. It is recommended that the designed shear stresses of PRC coupling beams should not exceed 12 MPa for concrete with cube compressive strength of 60 MPa.

**Figure 8:**
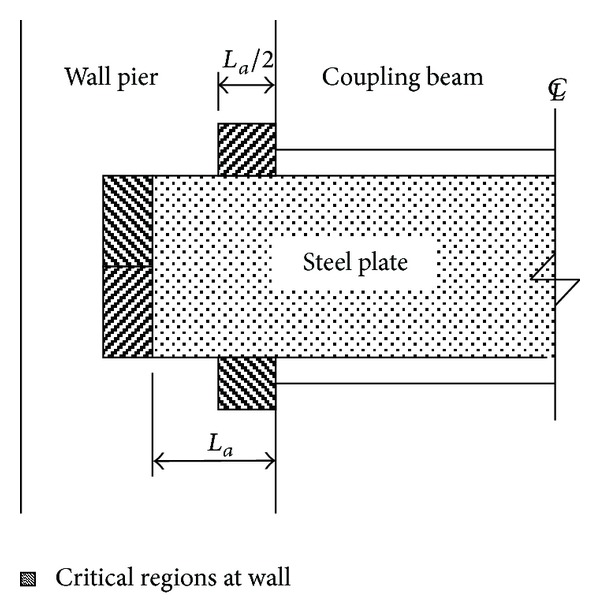
Critical regions prone to cracking at wall pier.

The critical regions in the wall piers were in areas where the plate anchor bore against the concrete. These areas include (1) areas above and below the plate anchors in the left and the right wall piers, respectively, basically concentrated in the first half of the anchor near the beam-wall joint, and (2) areas in contact with the upper half and the lower half of the vertical anchor edges in the left and the right wall piers, respectively. By considering the effect of reversed cyclic loads, the critical regions prone to cracking at the wall regions are depicted in Figure 8. Bearing provided to the vertical edges of the plate anchors became more important as the span-to-depth ratio decreased.

**Figure 9:**
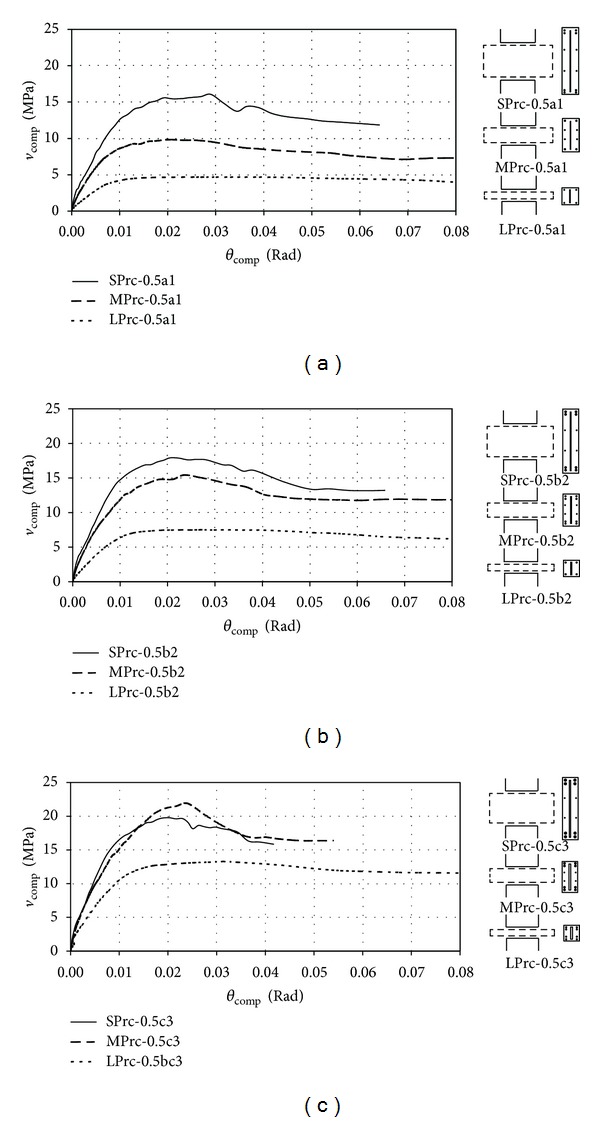
Computed shear stress-drift responses of models with (a) low, (b) moderate, and (c) high steel contents (*L*_*a*_ = 500 mm).

The effects of span-to-depth ratio and steel contents on the performances of PRC coupling beams are investigated.  Figure 9 shows the *v*_ comp _–*θ*_ comp_ relationships of three series of models with relatively low (series a1), moderate (series b2), and high (series c3) steel (including longitudinal reinforcement and steel plate) contents, respectively. Again, the three models in each series were of *L*_*a*_ = 500 mm, and the values of *ρ*_*s*_ and *t*_*p*_/*b* were constant. For LPrc units, ultimate shear strength was controlled by the flexural capacity of the beams. Yielding of longitudinal reinforcement of the beams and flexural inelastic deformation of the plates resulted in ductile failure modes. For MPrc and SPrc units, particularly with high steel ratios, failure of beams was controlled by the shear capacity of concrete; thus the beams failed in a brittle fashion.

### 3.2. Effects of Steel Ratio

Models of *L*_*a*_ = 500 mm from three different series with relatively low (series a1), moderate (series b2), and high (series c3) steel (including longitudinal reinforcement and steel plate) contents, respectively, are compared to investigate the effectiveness of the steel components in PRC coupling beams with different span-to-depth ratios. In each series, the values of *ρ*_*s*_ and *t*_*p*_/*b* were constant for the SPrc, MPrc, and LPrc models.  Table 2 shows the computed values of maximum shear strength (*V*_max⁡, comp _) and secant stiffness at yield (*k*_*y*, comp _), as well as the theoretical ultimate shear strengths (*V*_u_*) of all the models. The increase in capacity from low steel content to high steel content was the highest in the LPrc units (about 200%), but these models were still the least effective even with high steel content. This was reasonable as the contribution of the plate in resisting shear was limited by the plate bending capacity, which was governed by the available lever arm of a beam section for the internal resisting couples. Although a large lever arm was available for the internal resisting couples, the SPrc units were not necessarily the most effective when the steel content was further increased. In fact, the SPrc units experienced the smallest percentage increase in capacity from low steel content to high steel content. No more increase in shear strength could be observed when the steel content was increased from moderate to high. Moreover, all the SPrc units with thick steel plates of *t*_*p*_ = 36 mm could not develop their full capacities, and the problem is likely caused by insufficient wall reinforcement and will be discussed in Section 3.4.

The increase in capacity with the increase in steel content in the MPrc units was not as significant as in the LPrc units, but the former were the most effective under high steel content. However, the capacity was increased at a cost of decreased ductility in the MPrc units as the strength dropped rapidly after reaching the peak in Unit MPrc-0.5c3 with high steel content (see Figure 9(c)). The MPrc units (with span-to-depth ratio of 2) appear to be the most effective PRC coupling beams in terms of enhancement of strength under various steel ratios.

### 3.3. Effects of Anchorage Length of Steel Plate in Wall Pier

The anchorage length effect can be investigated by comparing the strengths and stiffness of models with identical beam geometry and beam steel ratios but different anchorage lengths (i.e., models in the same series of each group). The anchorage length could slightly affect the strength and stiffness of a PRC coupling beam, but the effect would diminish beyond the minimum required anchorage length for full capacity development. This explains why when the anchorage lengths in the MPrc units and the LPrc units were increased from 0.5*l* to 0.75*l* and from 0.375*l* to 0.5*l*, respectively, both the stiffness and the peak loads only increased insignificantly. This was in contrast with the cases when the *L*_*a*_ values were increased from 0.335*l* to 0.5*l* and 0.25*l* to 0.375*l*, respectively. The stiffness remained almost the same in the SPrc units for all the three anchorage lengths. This, on the one hand, suggests that an anchorage length of 0.5*l* may be good enough for the SPrc units and, on the other hand, suggests that the stiffness may be mainly determined by the beam geometry, and the role of the anchorage length diminishes as the span-to-depth ratio decreases.

The beam strengths increased with the anchorage length with a decreasing rate in all the SPrc and the LPrc units. Depending on the longitudinal steel ratio and the plate thickness, doubling the anchorage lengths in the SPrc and the LPrc units could cause an increase in strength ranging from 2 to 10%.

The response of the MPrc units was more sensitive to the change in anchorage length, and the strength increased more significantly with the increasing anchorage length in this group. No further increase in the shear strength as the increase in anchorage length could be observed in most units but not for units with high steel content. This suggests that the steel content may determine the minimum required anchorage length for full capacity development.

**Figure 10:**
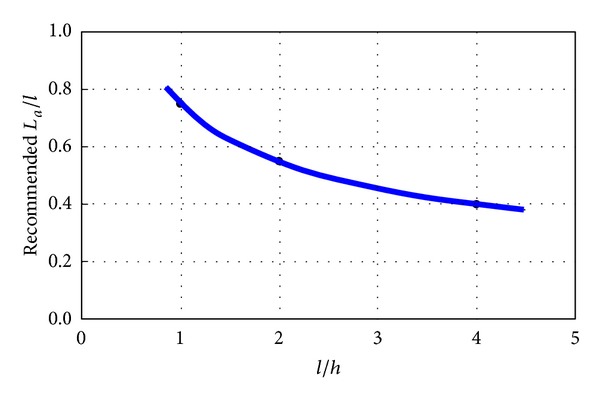
Recommended minimum *L*_*a*_/*l* value for preliminary design.

Based on the predicted variations of strength (as well as the stiffness) of PRC beams as well as the intensity of bearing stresses of shear studs at the anchorage regions, an empirical parabolic *L*_*a*_/*l*–*l*/*h* relationship (4) as shown in Figure 10 is recommended for preliminarily determining the minimum anchorage length required. The equation should be good enough for normal combinations of plate thicknesses and steel ratios when the material strengths are similar to the ones used in this study:

(4) $$\begin{array}{c} \frac{L_{a}}{l}=0.03{\left(\frac{l}{h}\right)}^{2}-0.27\left(\frac{l}{h}\right)+1\;\text{for}\;1.0\leq \frac{l}{h}\leq 4.0. \end{array}$$

It is noted that when the span-depth ratios *l*/*h* equal to 1, 2, and 4, the corresponding *L*_*o*_/*l* ratios are 0.76, 0.58, and 0.4, respectively. By adopting these *L*_*o*_/*l* ratios, it can be found from Table 2 that most of the computed shear strengths *V*_max⁡, comp_ are higher than the corresponding theoretical design shear strength *V*_u_*, except the ones with short span (SPrc units) combined with thick steel plates. The reasons for causing insufficient strength of PRC coupling beams will be discussed in the next section.

### 3.4. Effects of Wall Reinforcement Ratio

**Figure 11:**
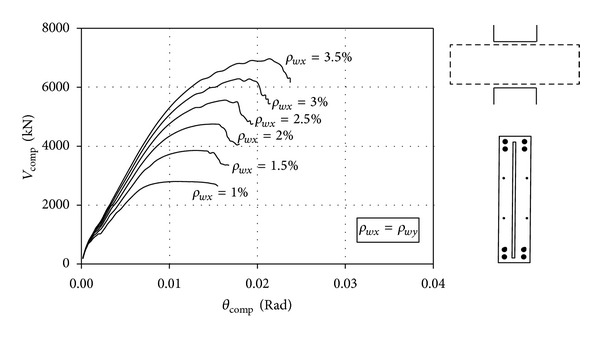
Computed load-drift responses of Unit SPrc-1.0c3 with different wall reinforcement ratios.

It has been shown in Table 2 that all the SPrc units with thick steel plates of *t*_*p*_ = 36 mm could not develop their full capacities (*V*_max⁡, comp _/*V*_u_* < 1), and the problem was likely caused by insufficient wall reinforcement. In order to investigate how much wall reinforcement would be required for Unit SPrc-1.0c3, the wall reinforcement ratios were varied in this model, and the computed load-drift responses are presented in Figure 11. This model with a plate anchorage length of 1.0*l* was chosen for the investigation as it was unlikely that its premature failure was associated with insufficient anchorage length. For simplification, the wall piers were provided with the same percentage of reinforcement in the vertical and the horizontal directions, that is, *ρ*_*wx*_ = *ρ*_*wy*_. In real practice, due to high axial loads acting on wall piers, steel ratio in walls in the vertical direction (*ρ*_*wy*_) is often higher than that in the horizontal direction (*ρ*_*wx*_).

The increase in beam strength with the increase in the wall reinforcement ratio confirms that the premature failures of the SPrc units with thick plates were caused by insufficient wall reinforcement. It can be observed that the beams can resist more loadings as the increase in the steel ratio *ρ*_*wx*_, and the beam strength will probably increase further when more wall reinforcement is provided. However, it is impractical to further increase the wall reinforcement ratio because of steel congestion. In fact, *ρ*_*wx*_ = 1.8% is already a rather high steel ratio for the walls. Hence the maximum shear stress of PRC coupling beams should not exceed 15 MPa. With a partial safety factor *ϕ* = 1.25, the design shear stress should be limited to 12 MPa (=15/1.25 MPa).

Together with the increase in beam strength, the beam rotation corresponding to the maximum loads increased steadily from about 0.01 Rad with 1% wall reinforcement to about 0.02 Rad with 3.5% wall reinforcement. Such a relatively large beam rotation may be too high for the building subjected to ultimate wind loading conditions.

## 4. Conclusions

 By employing a nonlinear finite element analysis that has been validated by the results of a previous experimental study, the effects of beam geometries and anchorage arrangements on the overall performances of PRC coupling beams have been studied numerically and presented in this paper. The findings from the parametric study are summarized as follows.

1. PRC coupling beams would be most effective with a span-to-depth ratio of about 2 under various longitudinal steel ratios.
2. The critical regions (above and below the plate anchors near the beam-wall joints and at the ends of the plate anchors), which are prone to cracking in the wall piers, have been identified.
3. Insufficient plate anchorage length would result in a reduction of beam strength and stiffness, but an increase of the anchorage length beyond the minimum required value for full capacity development would not further improve the beam performance much. A parabolic *L*_*a*_/*l*–*h*/*l* relationship is recommended for determination of the anchorage length.
4. Apart from insufficient plate anchorage length, insufficient wall reinforcement could also result in premature beam failure. However, the required wall reinforcement ratio could be far exceeding the practical limit in PRC coupling beams designed for extremely large shear stresses. The maximum allowable shear stress should therefore be limited to 15 MPa. In practice, it is more desirable for the plate to share about 50% of the total load resistance.
5. The results in the present study have enhanced the development of a comprehensive design procedure for the PRC coupling beams [18].

## Figures and Tables

**Table 1:** Reinforcement details of prototype coupling beams modeled in numerical study.

Group	Types (3 units each)	*V*_u_*(MPa)	Longitudinal rebars *A*_*s*_	Transverse rebars *A*_*sv*_	Plate size (mm) *h*_*p*_ × *t*_*p*_	Wall reinforcement
SPrc (*l/ h* = 1)	a1	11.6	4T20	T12-85-S.S.	920 × 12	Horizontal rebar: T20-150-E.F. Vertical rebar: T16-150-E.F.
	a2	11.8	2T32 + 2T25			
	a3	12.1	4T40			
	b1	15.0	4T20	T10-70-S.S.	920 × 20	
	b2	15.2	2T32 + 2T25			
	b3	15.5	4T40			
	c1	24.4	4T20	T12-100-S.S.	920 × 36	
	c2	24.6	2T32 + 2T25			
	c3	24.9	4T40			

MPrc (*l/ h* = 2)	a1	9.3	2T20	T12-85-S.S.	420 × 12	Horizontal rebar: T20-150-E.F. Vertical rebar: T16-150-E.F.
	a2	9.6	4T20			
	a3	9.6	2T32 + 2T25			
	b1	11.1	2T20	T10-70-S.S.	420 × 20	
	b2	11.4	4T20			
	b3	11.4	2T32 + 2T25			
	c1	16.9	2T20	T12-85-S.S.	420 × 36	
	c2	17.1	4T20			
	c3	17.4	2T32 + 2T25			

LPrc (*l/ h* = 4)	a1	7.8	4T10	T12-85-S.S.	200 × 12	Horizontal rebar: T20-150-E.F. Vertical rebar: T16-175-E.F.
	a2	8.1	2T20			
	a3	8.4	4T20			
	b1	8.3	4T10	T10-70-S.S.	200 × 20	
	b2	8.6	2T20			
	b3	9.0	4T20			
	c1	10.7	4T10	T10-80-S.S.	200 × 36	
	c2	11.0	2T20			
	c3	11.4	4T20			

*Notes*. S.S.: single stirrup; E.F.: each face.

**Table 2:** Calculated strength and stiffness for the prototype coupling beams.

Model	*V*_ max,comp_ (kN)			*k*_*y*,comp_ (MN/Rad)			*V*_u_* (kN)			*V*_ max,comp_/*V*_u_*		
	1	2	3	1	2	3	1	2	3	1	2	3
SPrc-0.5a	3500	3800	3900	246	278	300	2600	2600	2700	1.3	1.5	1.4
SPrc-0.5b	3500	3800	3900	256	290	316	3200	3300	3300	1.1	1.2	1.2
SPrc-0.5c	3500	3800	3900	276	303	314	4900	4900	5000	0.7	0.8	0.8
SPrc-0.715a	3700	3900	4000	256	290	308	2600	2600	2700	1.4	1.5	1.5
SPrc-0.715b	3800	3900	4000	279	307	336	3200	3300	3300	1.2	1.2	1.2
SPrc-0.715c	3900	3900	4000	313	321	353	4900	4900	5000	0.8	0.8	0.8
SPrc-1.0a	3900	4000	4000	274	292	319	2600	2600	2700	1.5	1.5	1.5
SPrc-1.0b	4000	4000	4000	303	326	356	3200	3300	3300	1.3	1.2	1.2
SPrc-1.0c	4000	4000	4000	336	362	377	4900	4900	5000	0.8	0.8	0.8

MPrc-0.335a	1060	1290	1580	85	94	107	900	1000	1100	1.2	1.3	1.4
MPrc-0.335b	1330	1530	1800	84	91	108	1200	1200	1200	1.1	1.3	1.5
MPrc-0.335c	1330	1660	1880	90	102	114	1700	1700	1700	0.8	1.0	1.1
MPrc-0.5a	1090	1320	1600	96	102	115	900	1000	1100	1.2	1.3	1.5
MPrc-0.5b	1400	1650	1880	100	105	118	1200	1200	1200	1.2	1.4	1.6
MPrc-0.5c	1800	1950	2180	108	119	125	1700	1700	1700	1.1	1.1	1.3
MPrc-0.75a	1120	1370	1620	97	106	119	900	1000	1100	1.2	1.4	1.5
MPrc-0.75b	1470	1700	1950	111	116	129	1200	1200	1200	1.2	1.4	1.6
MPrc-0.75c	2050	2260	2480	120	127	135	1700	1700	1700	1.2	1.3	1.5

LPrc-0.25a	230	290	390	17	21	26	200	250	350	1.2	1.1	1.1
LPrc-0.25b	320	370	460	22	25	28	270	330	430	1.2	1.1	1.1
LPrc-0.25c	460	500	600	24	27	30	400	450	520	1.1	1.1	1.1
LPrc-0.375a	240	290	390	20	23	28	200	250	350	1.2	1.2	1.1
LPrc-0.375b	330	370	470	25	27	32	270	330	430	1.2	1.1	1.1
LPrc-0.375c	470	510	610	32	34	37	400	450	520	1.2	1.1	1.2
LPrc-0.5a	240	290	390	21	24	28	200	250	350	1.2	1.2	1.1
LPrc-0.5b	330	370	470	26	28	32	270	330	430	1.2	1.1	1.1
LPrc-0.5c	470	510	610	32	33	39	400	450	520	1.2	1.1	1.2
